# B Cell Subsets as Severity-Associated Signatures in COVID-19 Patients

**DOI:** 10.3389/fimmu.2020.611004

**Published:** 2020-12-03

**Authors:** Víctor A. Sosa-Hernández, Jiram Torres-Ruíz, Rodrigo Cervantes-Díaz, Sandra Romero-Ramírez, José C. Páez-Franco, David E. Meza-Sánchez, Guillermo Juárez-Vega, Alfredo Pérez-Fragoso, Vianney Ortiz-Navarrete, Alfredo Ponce-de-León, Luis Llorente, Laura Berrón-Ruiz, Nancy R. Mejía-Domínguez, Diana Gómez-Martín, José L. Maravillas-Montero

**Affiliations:** ^1^Red de Apoyo a la Investigación, Universidad Nacional Autónoma de México e Instituto Nacional de Ciencias Médicas y Nutrición Salvador Zubirán, Mexico City, Mexico; ^2^Departamento de Biomedicina Molecular, Centro de Investigación y de Estudios Avanzados del Instituto Politécnico Nacional, Mexico City, Mexico; ^3^Departamento de Atención Institucional Continua y Urgencias, Instituto Nacional de Ciencias Médicas y Nutrición Salvador Zubirán, Mexico City, Mexico; ^4^Departamento de Inmunología y Reumatología, Instituto Nacional de Ciencias Médicas y Nutrición Salvador Zubirán, Mexico City, Mexico; ^5^Facultad de Medicina, Universidad Nacional Autónoma de México, Mexico City, Mexico; ^6^Departamento de Infectología, Instituto Nacional de Ciencias Médicas y Nutrición Salvador Zubirán, Mexico City, Mexico; ^7^Unidad de Investigación en Inmunodeficiencias, Instituto Nacional de Pediatría, Mexico City, Mexico

**Keywords:** B cells, DN B cells, transitional B cells, memory B cells, COVID-19

## Abstract

**Background:**

SARS-CoV-2 infection represents a global health problem that has affected millions of people. The fine host immune response and its association with the disease course have not yet been fully elucidated. Consequently, we analyze circulating B cell subsets and their possible relationship with COVID-19 features and severity.

**Methods:**

Using a multiparametric flow cytometric approach, we determined B cell subsets frequencies from 52 COVID-19 patients, grouped them by hierarchical cluster analysis, and correlated their values with clinical data.

**Results:**

The frequency of CD19^+^ B cells is increased in severe COVID-19 compared to mild cases. Specific subset frequencies such as transitional B cell subsets increase in mild/moderate cases but decrease with the severity of the disease. Memory B compartment decreased in severe and critical cases, and antibody-secreting cells are increased according to the severity of the disease. Other non-typical subsets such as double-negative B cells also showed significant changes according to disease severity. Globally, these differences allow us to identify severity-associated patient clusters with specific altered subsets. Finally, respiratory parameters, biomarkers of inflammation, and clinical scores exhibited correlations with some of these subpopulations.

**Conclusions:**

The severity of COVID-19 is accompanied by changes in the B cell subpopulations, either immature or terminally differentiated. Furthermore, the existing relationship of B cell subset frequencies with clinical and laboratory parameters suggest that these lymphocytes could serve as potential biomarkers and even active participants in the adaptive antiviral response mounted against SARS-CoV-2.

## Introduction

Over the past few years, the world suffered two different threatening outbreaks of coronavirus infection, causing Severe Acute Respiratory Syndrome (SARS) in 2002 and the Middle East Respiratory Syndrome (MERS) in 2011. In these outbreaks, both causative zoonotic pathogens, named as SARS-CoV and MERS-CoV respectively, were newly identified Betacoronavirus. By the end of 2019, an independent outbreak of a disease that generates respiratory-related symptoms was firstly reported in Wuhan, China, thus emerging as a new clinical entity now officially known as Coronavirus Disease 2019 (COVID-19) that subsequently spread globally. Rapidly, this illness’s causative agent was identified as an emerging coronavirus by genome full-sequencing approaches and later named SARS-CoV-2. Phylogenetic analysis of full-length genome sequences of SARS-CoV-2 showed that this virus is highly related to SARS-CoV and more distant from and less related to MERS-CoV (1).

Even though most COVID-19 cases are presented as asymptomatic or display only mild symptoms, patients’ remainder develops severe or even critical presentations (2, 3). The most frequent manifestations of COVID-19 include fever, asthenia, myalgia, olfactory and gustatory dysfunction, the most typical respiratory symptoms such as sore throat, cough, and dyspnea, and even enteric symptoms with abdominal pain, vomiting, and diarrhea (2–6). The major complication of severe COVID-19 is acute respiratory distress syndrome (ARDS) with dyspnea and respiratory failure that requires mechanical ventilation (7). Also, severe COVID-19 has been associated with cardiovascular complications, such as cardiomyopathies, arrhythmias, and heart failure, with coagulopathy presenting as thrombosis in different organs (8–10). Moreover, several hospitalized severe and critical COVID-19 patients exhibit high-levels of cytokines and chemokines such as IL-2, IL-6, IL-7, IL-10, G-CSF, CCL2, CCL3, CXCL10, and TNFα that seems to be associated with lymphopenia and the cytokine storm (that could derive in shock, organ failure, and potential death), both observed in SARS and MERS severe cases, thus suggesting a significant role of these features in the pathogenesis of COVID-19 (11, 12). In order to mount this antiviral response, which typically induces a type I IFN signature, innate immune cells are the first responders that get activated after infection, recognizing the virus pathogen-associated molecular patterns (PAMPs). For coronavirus, it is well-known that genomic RNA or the intermediate molecules generated during viral replication such as dsRNA are recognized by either endosomal RNA receptors (TLR3 and TLR7) or cytosolic RNA sensors such as RIG-I/MDA5 (13–15). Interestingly, for SARS-CoV and MERS-CoV, the innate response mediated by type I IFN is sometimes suppressed, particularly in severe disease cases, since these viruses use different signaling pathways-blocking mechanisms (16).

Regarding the adaptive immune system’s participation, T-cell responses against the SARS-CoV-2 spike protein have been recently characterized and correlate with either IgG and IgA antibody titers in COVID-19 patient cohorts, both relevant features for vaccine development and long-term (memory) immune responses (17–19). It is recognized that the Th1 response performs a leading role in viral infections. Cytokines and chemokines secreted by antigen-presenting cells (APCs) determine the effector fate of T cell subsets. Concomitantly, the humoral immune response mainly centered on producing neutralizing antibodies by plasma cells is expected to play a protective function by controlling infection at later disease stages and maybe preventing future re-infections (20). However, current reports do not exhibit a description or a role for B cell subsets during acute COVID-19.

B cells’ ability to differentiate into plasma cells that produce a vast antibody repertoire that protects against almost every potential pathogen, and at the same time, the generation of immunological memory available to suppress subsequent infections, represent the most recognized functions of these lymphocytes (21). However, B cells have also been implicated in pathogenic roles in autoimmune diseases, transplant, cancer, or even infections, exerting alternative antibody-independent responses that often rely on cytokine secretion with either, pro- or anti-inflammatory properties (22). Circulating human B cells are typically classified into different compartments that include immature recent bone marrow emigrants (transitional B cells), mature lymphocytes never-stimulated by their cognate antigen (naïve B cells), and terminally differentiated cells that resulted from previous immune responses due to antigen exposure (memory B cells and antibody-secreting plasmablasts/plasma cells); all of them with derived phenotypical and functional divergent subsets (22). Recently, the development of new powerful multiparametric flow cytometric and single-cell genomic approaches, has allowed the identification of emerging B cell populations with distinctive phenotypical and functional characteristics (23–25). Since many of these cells have been implicated in the pathogenesis of acute and chronic viral infections (22), we become interested in mapping them in COVID-19 patients, trying to find a link between their presence and the clinical presentations of this globally relevant disease.

## Materials and Methods

### COVID-19 Patients and Healthy Donors

COVID-19 patients and healthy donors were recruited at a tertiary care center in Mexico City (Instituto Nacional de Ciencias Médicas y Nutrición Salvador Zubirán). All patients with symptoms suggestive of COVID-19 and confirmed by a positive PCR test for SARS-CoV-2 on nasopharyngeal swab were invited to participate in the study. Upon patient admission, EDTA whole-blood samples for cellular analyses were taken. The severity of the disease was classified, also upon admission, as follows: Mild/Moderate illness: Fever, signs of airway disease, with or without a tomographic image indicating pneumonia. Severe illness: Any of the following: respiratory failure, respiratory rate >30 bpm, oxygen saturation < 92% at rest, arterial partial pressure of oxygen (PaO_2_)/fraction of inspired oxygen (FiO2) (PaO_2_/FiO_2_) ratio < 300 mmHg. Critical illness: Any of the following: requirement for mechanical ventilation, shock, or concomitant organ failure (26). All healthy controls (7 recruited) and patients signed an informed consent form before inclusion, and the institutional ethics and research committees approved the protocol (Ref. 3341) in compliance with the Helsinki declaration.

### Patients-Data Collection

Demographic and clinical data of patients were collected (**Table 1**), including gender, age, days of symptoms onset, symptoms (cough, headache, fever, dyspnea, arthralgia, myalgia, sore throat, nasal/conjunctival congestion, nausea or vomiting, diarrhea, and fatigue), vital signs including mean arterial pressure, heart rate, respiratory rate, oxygen saturation (SO_2_) and temperature, history of smoking and comorbidities (overweight/obesity, diabetes, hypertension, cardiopathy, cerebrovascular disease, chronic kidney disease, liver disease, immunodeficiency, or autoimmunity).

**Table 1:** Features of Coronavirus Disease 2019 (COVID-19) patients and healthy individuals.

Characteristic	All patients (n=52) Healthy subjects(n=7)	Mild/Moderate (n=19)	Severe (n=16)	Critical (n=17)
**Gender – #. (%)**				
Female Male	20 (38) 32 (62)	10 (53) 9 (47)	7 (44) 9 (56)	3 (18) 14 (82)
**Age in years – median (range)**	46 (25–80)	39 (25–68)	48 (25–68)	52 (29–80)
**Diagnosis of SARS-COV-2 (%)**				
Positive PCR from nasopharyngeal swab	52 (100)	19 (100)	16 (100)	17 (100)
**Days of symptoms – median (range)**	8 ±5.2 (1–30)	5 ±2.9 (1–10)	9 ±5.1 (3–22)	10 ±6.0 (5–30)
**Symptoms – # (%)**				
Cough Headache Fever Dyspnea Arthralgia Myalgia Sore Throat Nasal congestion Conjunctival congestion Nausea or vomiting Diarrhea Fatigue	42 (81) 30 (58) 43 (83) 31 (60) 30 (58) 34 (65) 17 (33) 15 (29) 5 (10) 0 (0) 11 (21) 27 (52)	14 (74) 14 (74) 14 (74) 5 (26) 8 (42) 10 (53) 6 (32) 5 (26) 3 (16) 0 (0) 2 (10) 9 (47)	13 (81) 8 (50) 16 (100) 11 (69) 11 (69) 11 (69) 6 (37) 5 (31) 1 (6) 0 (0) 3 (19) 8 (50)	15 (88) 8 (47) 13 (76) 15 (88) 11 (65) 13 (76) 5 (29) 5 (29) 1 (6) 0 (0) 6 (35) 10 (59)
**Vital signs – median**				
Mean arterial pressure Heart rate Respiratory rate SO_2_ Temperature	90 ±11.5 102 ±20.7 25 ±8.2 86 ±12.3 37.2 ±0.7	91 ±10.6 94 ±20.9 20 ±3.7 95 ±1.7 37.1 ±0.6	91 ±10.9 108 ±17.5 24 ±5.5 88 ±4.3 37.2 ±0.8	89 ±13.6 107 ±21.4 31 ±9.7 75 ±15.3 37.3 ±0.7
**Comorbidities – # (%)**				
Obesity Smoker Diabetes Hypertension Cardiopathy Cerebrovascular disease Chronic kidney disease Liver disease Immunodeficiency Autoimmunity Cancer	14 (27) 20 (38) 5 (10) 11 (21) 11 (21) 4 (8) 0 (0) 3 (6) 2 (4) 1 (2) 0 (0) 1 (2)	3 (16) 4 (21) 1 (5) 1 (5) 1 (5) 0 (0) 0 (0) 1 (5) 0 (0) 0 (0) 0 (0) 0 (0)	6 (37) 5 (31) 2 (12) 5 (31) 4 (25) 1 (6) 0 (0) 0 (0) 0 (0) 1 (6) 0 (0) 1 (6)	5 (29) 11 (65) 2 (12) 5 (29) 6 (35) 3 (18) 0 (0) 2 (12) 2 (12) 0 (0) 0 (0) 0 (0)
**Laboratory Values**				
White blood cell count Absolute lymphocyte count Hemoglobin, g/dl Platelet Count, K/µl Creatinine, mg/dl Total bilirubin, mg/dl Aspartate aminotransferase, U/L Alanine aminotransferase, U/L C-reactive protein, mg/dl Ferritin, ng/ml Lactate dehydrogenase, U/L D-dimer, ng/ml Troponin, pg/ml INR			7.0 ±2.3 831.7 ±559.1 15.2 ±1.4 208 ±76.8 0.8 ±0.2 0.6 ±0.1 49.6 ±34.1 50.8 ±40.4 54.1 ±173.2 783 ±892.5 347.4 ±160.4 990.9 ±1498 5.3 ±2.7 1.1 ±0.09	10.8 ±4.6 731 ±357.4 15.3 ±2.2 231 ±81.1 2.4 ±5.4 0.6 ±0.2 124.3 ±242.8 108.1 ±250.9 15.9 ±7.1 1747.7 ±3444.0 541.3 ±240.1 1127 ±796.7 257.7 ±570.8 1.2 ±0.7
**Treatments – # (%)**				
Hydroxychloroquine Azithromycin Antibacterial antibiotic Tocilizumab Remdesevir Oseltamivir Vasopressors Mechanical ventilation			14 (87) 16 (100) 11 (69) 2 (12) 2 (12) 13 (81) 0 (0) 0 (0)	9 (53) 13 (76) 14 (82) 3 (18) 1 (6) 8 (47) 10 (59) 11 (65)
**Measures of illness severity**				
qSOFA NEWS PSI-PORT			1 ±0.6 7 ±2.0 54 ±24.0	1 ±0.8 8 ±2.9 92 ±41.0
**Complications – # (%)**				
Shock Secondary Infections			0 (0) 1 (6)	3 (18) 8 (47)
**Gasometry Values**				
FiO_2_ pH Art PaO_2_ Art PaCO_2_ Art HCO_3_ Lactate PaO_2_/FiO_2_			27 ±13.1 7.43 ±0.09 70.0 ±30.5 30.5 ±3.1 21.8 ±1.7 1.3 ±0.6 271.5 ±78.4	48 ±22.0 7.42 ±0.09 63.4 ±28.0 30.1 ±3.2 19.8 ±3.5 2.1 ±1.6 163.2 ±113.8
**Outcomes – # (%)**				
Admission to hospital Death Recovery		0 (0) 0 (0) 19 (100)	16 (100) 0 (0) 16 (100)	15 (100) 9 (53) 4 (23)
**Healthy Individuals**				
Age	37 (32–41)			
**Gender**				
Female Male	3 4			

SO2, oxygen saturation; INR, International Normalized Ratio; qSOFA, Quick SOFA Score for Sepsis; NEWS, National Early Warning Score; PSI-PORT, Pneumonia Severity Index; FiO_2_, fraction of inspired oxygen; PaO_2_, partial pressure of oxygen; PaCO_2_, partial pressure of carbon dioxide; PaO_2_/FiO_2_, ratio of arterial oxygen partial pressure to fractional inspired oxygen.

For severe and critical patients only, routine laboratory tests were taken, including a complete blood count, glucose, blood urea nitrogen (BUN), creatinine (Cr), liver function tests, C-reactive protein (CRP), lactate dehydrogenase (LDH), creatinine kinase (CPK), fibrinogen, D-dimer, coagulation tests and ferritin. Therapeutic drug-use, respiratory support, complications (shock or secondary infections), and outcomes were documented. The following disease-severity scores were also calculated for these patients: qSOFA (quick Sepsis-related Organ Failure Assessment: identifies patients outside the ICU with a suspected infection that are at high risk for in-hospital mortality), NEWS (National Early Warning Score: determines the degree of illness and prompts critical care intervention), PSI-PORT (Pneumonia Severity Index/Pneumonia-patient Outcomes Research Team: calculates the probability of morbidity/mortality among patients with community-acquired pneumonia) (27).

### Multiparametric Flow Cytometry Analysis

**Figure 1:**
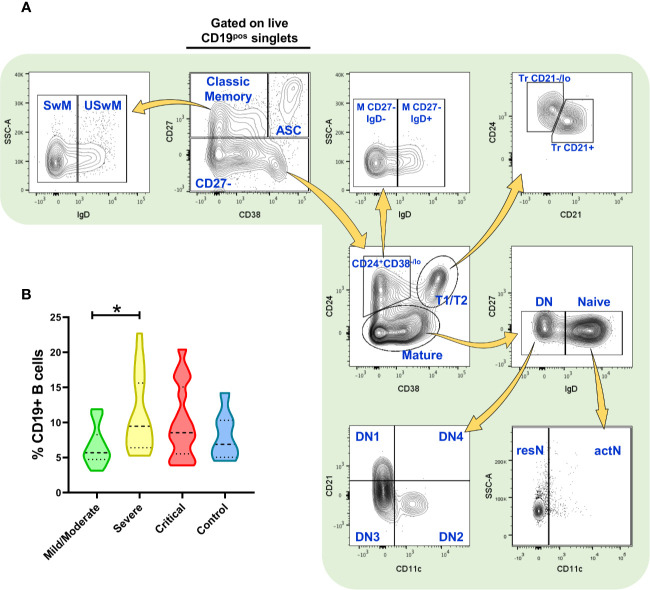
Flow cytometry analysis of B cell subsets and frequency of total (CD19^+^) B cells. **(A)** Gating strategy for the identification of the indicated B cell subsets in peripheral blood mononuclear cells (PBMCs) (depicting representative results from a healthy control) previously selected from singlets gate (SSC-A vs. SSC-H) and live Zombie Green^-^ cells. **(B)** Frequency of total CD19^+^ B cells in PBMCs from patients infected with SARS-CoV-2 (n=52) and healthy controls (n=7, negative PCR for SARS-CoV-2). Frequency values are displayed as mean (dashed line) plus lower and upper quartiles (dotted lines). The data were analyzed by a Kruskal-Wallis test followed by a Dunn’s *post-hoc* test. *p < 0.01. This figure was made using arrows from Servier Medical Art (http://smart.servier.com/), licensed under a Creative Commons Attribution 3.0 Unported License (https://creativecommons.org/licenses/by/3.0/).

After peripheral blood mononuclear cells (PBMCs) isolation by density gradients with Ficoll-Paque (GE Healthcare Life Sciences), cells were resuspended in RPMI-1640 with phenol red (Gibco) and counted. PBMCs staining was performed using conjugated monoclonal antibodies indicated in **Supplementary Table 1**; dead cells were excluded using the live/dead discrimination dye Zombie Green (BioLegend), as well as singlets discrimination with size and complexity parameters before cell analysis. Briefly, for extracellular staining, cells were treated with a human FcX blocker antibody mix (BioLegend) for 10 min. Without washing, cells were incubated for 30 min at 4°C with the antibody cocktail. Cells were then centrifuged at 1,500 rpm for 5 min and fixed with fixation buffer (BioLegend) for 1 h. Finally, cells were washed once with cell staining buffer (BioLegend) and then resuspended in 500 µl of the same buffer for immediate (or no more than a 12 h delay) flow cytometric analysis on a BD LSRFortessa using FACSDiva software (both from BD Biosciences). Up to 1x10^6^ cells were analyzed using FlowJo v10 software (BD Biosciences) with the strategy depicted in **Figure 1**, developed by using Fluorescence Minus One (FMO) controls to define gates. Compensation was assessed using CompBeads (BD Biosciences) and single stained fluorescent samples.

### Analysis of Variance and Multiple Comparison Post-Hoc Tests

Statistical differences of cell populations frequencies among patient groups of disease severity were evaluated by the Kruskal-Wallis test, followed by Dunn’s *post-hoc* test employing Prism 8 software (GraphPad).

### Hierarchical Clustering Analysis

Hierarchical clustering of both patients’ samples and cell populations frequencies determined by Ward’s method (Euclidean distance) to generate a heat-map with associated dendrograms. Ward’s minimum-variance linkage agglomeration clusters method joins clusters to maximize the likelihood at each level of the hierarchy by increase variance in within-group dispersion and displaying clear relationships among clusters. For this analysis, we excluded some of the cell subsets with repeated statistical information (correlation) within each “parental” population (memory, non-classical memory, Tr, DN, naïve) according to **Supplementary Figure 1**; these populations include the Tr CD21^-/lo^ that highly correlates with the very phenotypically-similar Tr CD21^+^, resN with their actN counterparts, and the DN4 subset that was also excluded since they were almost absent in every individual analyzed. The analysis and heat-map were generated in R, v. 4.0.2 (R Foundation for Statistical Computing, Vienna, Austria; URL http://www.R-project.org/).

### Correlation Analyses between B Cell Subsets and Clinical Features

Association between all B cell subset frequencies obtained and indicated severity indexes/clinical or laboratory variables were evaluated with Spearman’s correlation, with values graphically represented in the matrix of **Figure 4A**. The analyses, matrix, and graphs were performed in R, v. 4.0.2 (R Foundation for Statistical Computing, Vienna, Austria; URL http://www.R-project.org/).

## Results

### Circulating B Cell Diversity in COVID-19 Patients

Since ongoing vaccine development efforts are currently advancing, there is an urgent need to characterize immune reactions involving the establishment of protective responses, particularly concerning adaptive immunity. In this context, some reports detailing T cell contribution against SARS-CoV-2 have recently emerged (23, 28). However, although being the precursors of protective antibody responses, B cells have not yet received enough attention during the COVID-19 course. Consequently, we applied a simple multiparametric flow cytometry approach to identify and quantify some of the most relevant peripheral blood B cell subsets in 52 COVID-19 patients, clinically segregated into mild/moderate, severe, and critical groups.

Using our approach, we first segregate total CD19^+^ B cells into CD27^+^ CD38^-/lo^ “classic” memory B cells, CD27^+^ CD38^hi^ antibody-secreting cells (ASC)/plasmablasts, and a mixed subset consisting of CD27^-^ cells. Memory population were further subdivided into unswitched IgD^+^ (USwM) and switched IgD^-^ (SwM) B cells while CD27^-^ cell group was fractionated into three categories: transitional (Tr) 1 and 2 (T1/T2) CD24^+^ CD38^hi^ cells further subdivided into Tr CD24^hi^ CD21^-/lo^ (Tr CD21^-/lo^) and CD24^lo^ CD21^+^ (Tr CD21^+^); a non-classic CD27^-^ CD24^+^ CD38^-/lo^ that can also be segregated into IgD^+^ (M CD27^-^ IgD^+^) and IgD^-^ (M CD27^-^ IgD^-^) subpopulations; and finally, the “mature” CD24^-^ fraction that includes the naïve IgD^+^ CD38^-/+^ cells and the double negative B cells (DN, named as that by its CD27^-^ IgD^-^ phenotype). The naïve subset was additionally separated by CD11c expression into resting (CD11c^-^, resN) and activated (CD11c^+^, actN) while DN was fractionated into four subsets (DN1 to DN4) based on their CD11c and CD21 expression. All this strategy is depicted in **Figure 1A**, and the phenotypes obtained are defined in **Supplementary Table 2**.

Interestingly, just by analyzing the total CD19^+^ B cell fraction of PBMCs, we were able to detect differences in the frequency of these cells in COVID-19 patients when compared to healthy subjects, that although no significant, seem to be slightly increased particularly at severe and then critical cases (**Figure 1B**). When absolute cell numbers were analyzed, we did not detect significant differences in the B cell compartment between healthy subjects and COVID-19 patients of any category (**Supplementary Table 3**). Nonetheless, since humoral responses seem to play a role in the severity of COVID-19 (29, 30), we decided to assess diverse B cell subsets and, indeed we found striking differences in the frequencies of these B cell subpopulations.

### Altered Immature and Effector B Cell Compartments in COVID-19

Our analyses revealed not only the insignificant changes in total CD19^+^ B cells but also more profound alterations in specific subsets, particularly at the transitional and the terminally differentiated memory B cells and ASC compartments.

**Figure 2:**
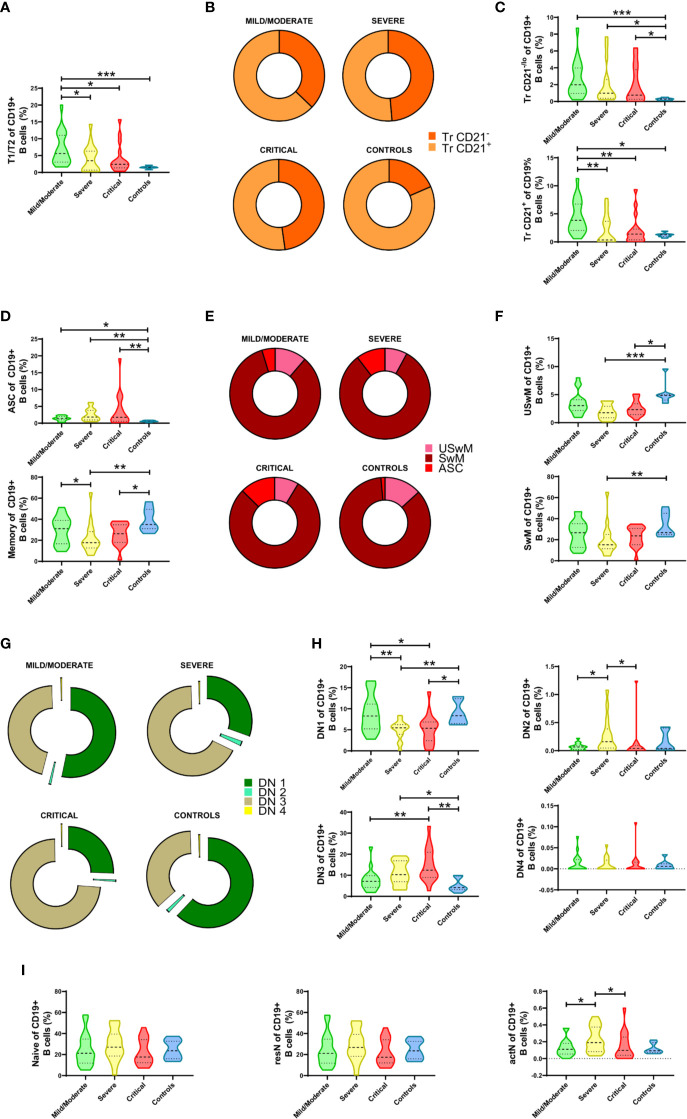
Alterations in the frequencies of peripheral B cell subsets from Coronavirus Disease 2019 (COVID-19) patients. **(A)** Comparative analysis of T1/T2 parental subset frequencies regarding CD19^+^ B cells. **(B)** Graphical representation of the Tr CD21^-/lo^ and Tr CD21^+^ mean frequencies in COVID-19 patients and controls. **(C)** Comparative analysis of Tr CD21^-/lo^ and Tr CD21^+^ frequencies relative to CD19^+^ B cells. **(D)** Comparative analysis of antibody-secreting cell (ASC) and Memory parental subsets relative to CD19^+^ B cells. **(E)** Graphical representation of the ASC, USwM, and SwM mean frequencies in COVID-19 patients and controls. **(F)** Comparative analysis of USwM and SwM subset frequencies relative to CD19^+^ B cells. **(G)** Representative composition of double negative (DN) fraction by graphing DN1, DN2, DN3, and DN4 subset mean frequencies. **(H)** Comparative analysis of DN1, DN2, DN3, and DN4 subset frequencies regarding CD19^+^ B cells. **(I)** Comparative analysis of parental naïve, resN, and actN subsets relative to CD19^+^ B cells. For all graphs, frequency values are displayed as mean (dashed line) plus lower and upper quartiles (dotted lines). The data were analyzed by a Kruskal-Wallis test followed by a Dunn’s *post-hoc* test. *p < 0.01, **p < 0.001. ***p < 0.0001.

Firstly, we observed a significant increase in the transitional T1/T2 cell fraction compared to its correspondent in healthy controls, which appeared more evident in mild/moderated cases but remained atypically expanded in severe and critical patients (**Figure 2A**). Once segregated according to their phenotype, we noticed a mean shift in this compartment’s composition with a more evident expansion in the Tr CD24^hi^ CD21^-/lo^ fraction (**Figure 2B**). When both Tr sub-compartments were independently analyzed, it becomes evident that both Tr CD21^-/lo^ and Tr CD21^+^ cells increment in COVID-19 patients and that their expansion is marked in mild/moderate cases but also remained in severe and critical courses (**Figure 2C**).

When the ASC subset was checked, we also detected a discrete increment of these cells in mild/moderate COVID-19 patients that was seen significantly magnified according to disease severity (**Figure 2D**, upper panel). In contrast, the COVID-19 memory compartment was seen significantly decreased, especially in severe and critical cases (**Figure 2D**, lower panel). When mean frequency values are compared, ASC subset enlargement is corroborated (**Figure 2E**), but memory cell population changes were more discrete. To overcome this limitation, we defined USwM and SwM subsets, and as depicted in **Figure 2F**, both were affected and predominantly reduced in severe and critical diseases.

Remarkably, among B cell compartments that showed one of the most outstanding alterations in response to SARS-CoV-2 is the DN (CD27^-^ IgD^-^) fraction. Except for the DN4 compartment found almost absent, mean frequencies of DN1, DN2, and DN3 subsets exhibited shifted distributions depending on the COVID-19 severity (**Figure 2G**). DN1 (CD21^+^ CD11c^-^) B cells showed no differences between healthy subjects and mild/moderate COVID-19 patients but exhibited a significant reduction in severe and critical cases; DN2 (CD21^-^ CD11c^+^) B cells only display a significant increase in severe vs. mild/moderate and critical cases while DN3 (CD21^-^ CD11c^-^) fraction was by far the most altered since their frequency was seen significantly increased as disease severity increased (**Figure 2H**).

Finally, while no differences were detected in the gross naïve B compartment, a significant increase in CD11c^+^ activated naïve (actN) lymphocytes was observed in severe cases compared to mild/moderate patients. Interestingly, this increment is only detectable in severe COVID-19 as critical patients display a reduction, rendering similar numbers than the mild/moderate cases. (**Figure 2I**).

Since most of these B cells subsets frequencies are age-dependent and recognizing that our available control group does not age-match so well with the study subjects, we performed and independent comparison analysis where only COVID-19 patients within the same age-range as the controls were selected, independently of their particular severity (due to sample size restrictions). As expected, this approach evidenced significant differences in the frequencies of most of the B cell subsets analyzed (initially displaying significant differences as indicated in **Figure 2**) between healthy controls and COVID-19 patients (**Supplementary Table 4**).

### The Frequencies of B Cell Subsets Are Associated With COVID-19 Severity Profiles

COVID-19 is currently acknowledged as a devastating disease for which clinical and conventional laboratory parameters have not been proven to reflect the disease state and outcomes accurately. Therefore, we aimed to address these B cell subsets as a more precise criterion of disease severity and clinical phenotype.

**Figure 3:**
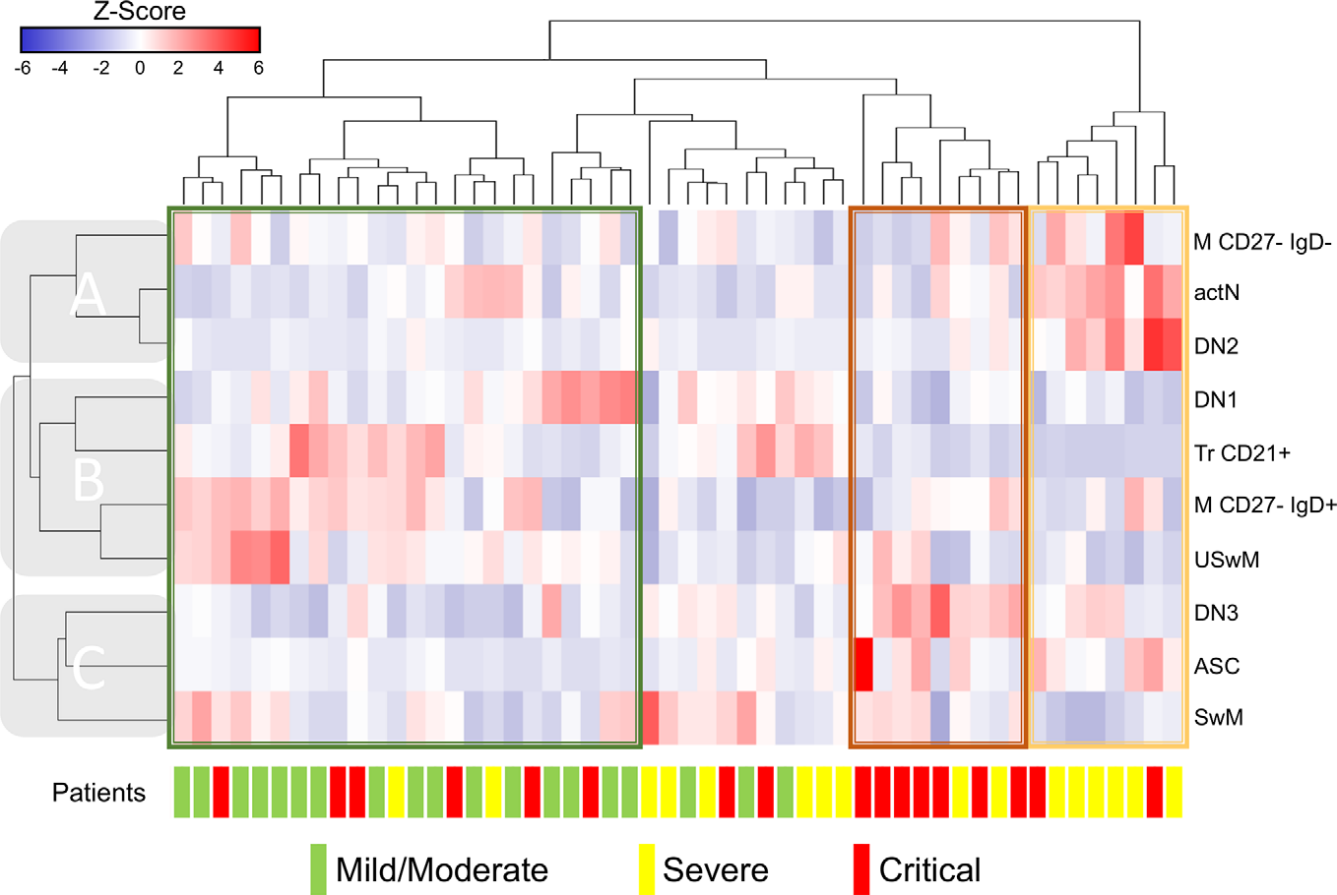
Clustering analysis from Coronavirus Disease 2019 (COVID-19) patients vs. B cells subsets. Hierarchical clustering analysis generated by Ward’s method relating mild/moderate (green, n= 19), severe (yellow, n= 16) and critical (red, n= 17) patients with low-correlating B subset frequencies depicted according to the score denoted in the upper color-scale bar. Associated horizontal dendrograms denote the patients clustering, standing out clusters containing mainly mild/moderate patients (enclosed in green), severe (enclosed in yellow) or critical (enclosed in red). Vertical dendrograms show three main subpopulation clusters **(A–C)**.

Since several B cell subsets frequencies showed significant alterations, we decided to group them by performing hierarchical clustering to identify profiles that could be associated with a particular COVID-19 presentation. Also, as stated in methods, we excluded the subsets that exhibited high-value correlation coefficients between them, according to **Supplementary Figure 1**. We then use the remaining ten different B cell subsets to generate the heat map in **Figure 3**; in this way, we could identify three primary horizontal clusters predominantly containing each of the COVID-19 presentations: mild/moderate enclosed in a green rectangle, severe in yellow or critical in red. As pointed in **Figure 3**, patient clusters are seen associated with B cell subsets (vertical) clusters: clade “A” shows an evident pattern where M CD27^-^ IgD^+^, actN, and DN2 cell frequencies stand out in severe patients cluster; a similar situation is detected for clade “C” where DN3, ASC, and SwM subsets seem to be increased in critical cases. Clade “B” subsets display a not so homogeneous pattern associated with mild/moderate cases; even though we can detect M CD27^-^ IgD^-^ and USwM populations highlighted in two sub-clusters and the Tr CD21^+^ and DN1 in other independent small sub-clusters containing primarily mild/moderate patients. In line with these enhanced patterns, it is possible to visualize the opposite reductions in some of the different clusters’ populations, thus profiling severity-associated signatures for the different COVID-19 patients.

### B Cell Subsets Correlate With Several Clinical and Laboratory Features of COVID-19

**Figure 4:**
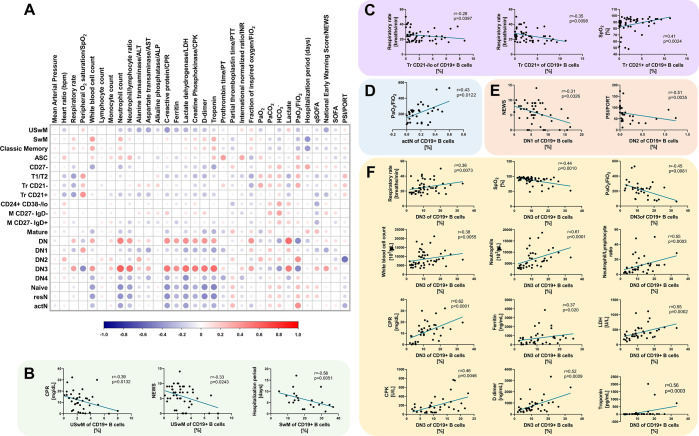
B cell subsets correlate with severity scores and different clinical parameters in Coronavirus Disease 2019 (COVID-19) patients. **(A)** Correlation matrix showing a graphical representation of calculated Spearman’s coefficient calculations between the B cell subset frequencies and indicated severity indexes/clinical and laboratory variables. The underlying color scale indicates Spearman’s coefficient values. The size of each dot also denotes the correlation strength. **(B)** Statistically significant correlations between memory B cell subsets and C-reactive protein (CRP), NEWS score and hospitalization length. **(C)** Statistically significant correlations between Tr B cell subsets and respiratory rate and SpO_2_. **(D)** The statistically significant correlation between actN subset and PaO_2_/FiO_2_. **(E)** Statistically significant correlations between DN1 B cell subset and NEWS score and DN2 subset with PSI/PORT score. **(F)** Statistically significant correlations between DN3 cell subset and respiratory rate, SpO_2_, PaO_2_/FiO_2_, white blood cell count, neutrophils, neutrophil/lymphocyte ratio, CPR, ferritin, lactate dehydrogenase (LDH), creatinine kinase (CPK), D-dimer, and troponin. In all graphs, calculated Spearman’s correlation (r) and significant p values (at least p < 0.01) are shown.

Since disease severity scales do not always reflect precisely the various abnormalities found in patients with COVID-19, we addressed the correlation coefficients between the B cell subsets and the clinical and laboratory features. The results obtained were then graphically represented in the matrix displayed in **Figure 4A**. Interestingly, ventilatory parameters, such as respiratory rate, SpO_2_, and PaO_2_/FiO_2_, showed significant correlation with immature (**Figure 4C**), activated naïve B cells (**Figure 4D**), as well as the DN3 subset (**Figure 4F**). Besides, memory B cells (**Figure 4B**) and DN1/DN2 subsets (**Figure 4E**) also negatively correlated with disease severity scores, such as NEWS and PSI/PORT. The only B cell subset that correlated with a clinical outcome, such as the hospitalization length, was the memory B cell subset, for which higher amounts of these cells correlated with a shorter hospitalization period. DN3 cells showed a broader significant correlation pattern with several features such as the before mentioned ventilatory parameters, including respiratory rate, oxygen saturation, and PaO_2_/FiO_2_. Furthermore, this particular B cell subset also displayed a positive correlation with different laboratory features, including leukocytes, neutrophils and the Neutrophil/Lymphocyte ratio, acute phase reactants, such as CRP and ferritin levels, markers of increased cellular turnover, including CPK and LDH as well as coagulopathy parameters, such as D-dimer and troponin.

## Discussion

Intriguingly, our COVID-19 patients displayed no significant differences in CD19^+^ peripheral blood B cell absolute numbers (**Supplementary Table 3**). Noteworthy, they showed slightly elevated relative frequencies of CD19^+^ B cells in peripheral blood, significantly increased between mild/moderate and severe patients (**Figure 1B**). These observations are of particular relevance since recent reports stand out the evidence of total or T cell lymphopenia in severe/critical COVID-19 patients (3, 11, 18, 26, 31); in this way, the B cell peripheral compartment in our cohort appears as refractory concerning to infection-induced lymphopenia, a trend that was also recently reported by other groups (32, 33). When specific B cell subsets were analyzed, absolute number comparisons rendered a few significant differences among COVID-19 patient groups (**Supplementary Table 3**). The same differences were observed as magnified when relative frequencies were instead analyzed. Hence, although no changes in total numbers of B cells could be seen, the proportions of some of the B cell subsets analyzed are being modified in response to the disease onset, thus making the comparison of subset relative frequencies more adequate for subsequent analyses.

Regarding the Tr B cells subset, it has been reported that the inflammatory context of different infectious or autoimmune diseases promotes the expansion of this compartment (34–37). Accordingly, our data show that the percentage of Tr B cells of the three groups of COVID-19 patients significantly increased compared to healthy donors. These Tr B cells can be segregated into two subsets: Tr CD21^-/lo^ cells and Tr CD21^+^, both observed as altered in our study since the three groups of COVID-19 patients exhibited a similar expansion. Although typically defined as “immature”, recent evidence support a direct role for human Tr cells in protection against infections by differentiation into ASC, mediated by pattern recognition receptors (38); therefore, the Tr expansion observed here, and also other COVID-19 latest studies (39, 40), could be explained as an “innate-like” response against SARS-CoV-2, requiring further characterization.

Furthermore, ASC frequencies also increased considerably in the three patient categories, showing a significant difference compared with healthy controls. The relevance of abnormally ASC numbers in response to COVID-19 presentation is unclear, although several reports have addressed their importance for the early production of high levels of SARS-CoV-2 specific antibodies (31, 41). Some studies have shown a worsening clinical status in other viral infections like dengue, that correlated with increased frequency of specific plasmablasts (42). Also, an increase in the number of ASC could result from the rapid differentiation of the already expanded Tr population, as suggested in other recent COVID-19 studies (39, 43).

On the other hand, the percentages of total memory B cells in the three COVID-19 groups of patients were low compared to healthy donors. Severe and critical groups presented a decrease in USwM B cells while only severe patients exhibit a significant decrease in SwM, both cases compared to healthy donors. These observations reproduce what is documented in other COVID-19-related studies (39, 40, 44, 45), proposing that this memory-decline could be the result of the activation of pre‐existing memory cells (specific for a coronavirus different from SARS‐CoV‐2), differentiating into “atypical” memory (CD27^-^) and/or ASC.

Additionally, total naïve B cells of COVID-19 patients and their subpopulations resN (CD11^-^) and actN (CD11c^+^), did not show significant differences compared to healthy donors. Only the severe group significantly increased actN B cells population compared to the mild/moderate and critical groups, in an expected response due to the hyper-inflammation in these patients, possibly inducing B cell activation processes. Our data might suggest that CD11c expression is modulated by a fine-tuning inflammatory threshold, which is overpassed in critical disease, similar to what is seen in activated memory CD11c^+^ B cells (46).

Finally, although the percentage of the total DN population in the three groups of COVID-19 patients did not present significant differences compared to healthy donors, three subpopulations indeed showed significant changes: DN1 cells decreased in all three patient groups, while DN2/DN3 seems to be increased, particularly in the severe and/or critical groups. Among these clinical categories, the DN1:DN3 ratio was reversed. To understand the importance of these intriguing findings, the still unknown function of each subpopulation of DN B cells needs to be fully elucidated, both in health and disease conditions, but mainly related to infectious processes.

Since we found several circulating B subset alterations, our data suggest that this approach could identify patterns or signatures associated with any disease severity groups. Accordingly, the hierarchical clustering analysis shows that most critical COVID-19 patients aberrantly display high proportions of ASC, DN3, and SwM B cells in a similar trend that severe patients exhibited high proportions of DN2, atypical CD27^-^ switched memory, and actN. Although maybe related to disease development, these alterations’ functional implications could not yet be fully explained besides the expected expansion of the actN, ASC, and memory compartments due to the infectious process itself and the potential pro-inflammatory role assigned to the DN2 subset (47). Regarding the more heterogeneous mild/moderate associated clusters, a similar explanation could apply to the expansion of the memory subsets seen here. However, concerning the Tr and DN1 subsets associated with some specific clusters where mild/moderate patients stand out, suggest that these cells could develop protective regulatory functions that have been described for the Tr B cells before (48), thus potentially interfering with the severe disease onset. According to our analysis, this possibility could also be supported by the fact that these specific populations are shown as relatively less represented in the severe and critical presentations than in the mild/moderate ones.

Additionally, our data indicate that these B cell subsets are highly correlated with the clinical phenotype in COVID-19. In this regard, it has been acknowledged that a hypoxic extracellular environment is present in immunological niches during normal development and in pathological conditions, including infections (49). Under these circumstances, hypoxia can regulate lymphocyte development in a HIF-1 dependent manner (50). Therefore, the correlation between different B cell subsets and ventilatory parameters, particularly oxygen saturation and PaO_2_/FiO_2_, could be related to the hypoxic conditions during COVID-19 infection. Particularly, since we found that lower levels of SaO_2_ and PaO_2_/FiO_2_ correlated with higher counts of DN3 cells, it might be feasible, that indeed under this hypoxic environment, the development program tilts toward the DN3 subset. Besides, we found that higher memory B cell counts correlated with lower risk scores (i.e., NEWS, PSI/PORT) as well as a shorter hospitalization period. These data are in agreement with those reported by Juno J, et al., who showed that COVID-19 recovered patients indeed displayed strong humoral responses dependent on memory B cells specific to the viral “spike” glycoprotein S (51). Interestingly, the DN3 subset, which, as described before, is entirely novel and not yet characterized, was the one that showed more correlation with several laboratory features that have been related to critical COVID-19 (52–54). In agreement with our findings, the DN3 subset has recently been reported to be expanded in critical COVID-19 patients and related to activated naïve B cells and the DN2 subset by hierarchical clustering (55). As shown by Woodruff M., et al., the presence of DN3 was related to a predominant extrafollicular B-cell response, which implies an earlier production of antibodies with low-affinity maturation, memory formation, and unknown antigen specificity (55). Nonetheless, our present work still represents the first report on the correlation of this novel B cell subset with clinical features of COVID-19.

We are aware that our study has several limitations. Among them is the limited sample size for elderly healthy donors since several B cell subsets are age dependent. However, the inclusion of this high-risk population is not feasible since most of them are still confined. Nonetheless, we found similar results on the age-paired analysis, which must be validated in this elderly population. Additionally, in our cohort, we could not measure the length of the incubation period, and we must acknowledge that this variable could influence some of the B cell subset frequencies (i.e., Tr B cells). Besides, our study’s unicentric and cross-sectional design does not allow us to fully address the prognostic value of these B cell subsets in COVID-19, which should be further validated.

## Data Availability Statement

The raw data supporting the conclusions of this article will be made available by the authors, without undue reservation.

## Ethics Statement

The studies involving human participants were reviewed and approved by Institutional Ethics and Research Committees (Ref. 3341); Instituto Nacional de Ciencias Médicas y Nutrición Salvador Zubirán. The patients/participants provided their written informed consent to participate in this study.

## Author Contributions

VS-H and JT-R contributed equally to the design and performance of experiments, analysis, and interpretation of data. RC-D and SR-R performed experiments and analyzed data. JP-F and DM-S assisted in processing and preservation of patient samples. GJ-V assisted with flow cytometric procedures. AP-F collected patient data, generated, and organized our clinical database. VO-N, AP-d-L, LL, and LB-R assisted in writing and edited the manuscript. NM-D performed bioinformatics and statistical analyses. DG-M and JM-M designed and performed experiments, supervised general work, wrote, and edited the manuscript. All authors contributed to the article and approved the submitted version.

## Funding

This work was supported by Consejo Nacional de Ciencia y Tecnología [Grants A3-S-36875, F0005-2020-01-313252] and UNAM-DGAPA-PAPIIT Program [Grants IN213020, TA101320].

## Conflict of Interest

The authors declare that the research was conducted in the absence of any commercial or financial relationships that could be construed as a potential conflict of interest.
