# Supplementary material for: B Cell Subsets as Severity-Associated Signatures in COVID-19 Patients
Source: Front Immunol. 2020 Dec 3;11:611004. doi: 10.3389/fimmu.2020.611004 (PMC7744304; doi:10.3389/fimmu.2020.611004)
Supplement: Supplementary file 1 [file DataSheet_1.pdf]

Supplementary Material

Supplementary Table 1. Antibodies and flow cytometry reagents used for flow cytometry analysis.

Antibody/reagent	Manufacturer	Catalog number
BUV496 anti-human CD19	BD Horizon	564656
APC/Cy7 anti-human CD27	BioLegend	302816
PerCP/Cyanine5.5 anti-human IgD	BioLegend	348208
Brilliant Violet 421 anti-human CD24	BioLegend	31122
Brilliant Violet 650 anti-human CD38	BioLegend	356620
Alexa Fluor 700 anti-human CD21	BioLegend	354918
PE/Dazzle 594 anti-human CD11c	BioLegend	301642
Zombie Green fixable viability kit	BioLegend	423112

Supplementary Table 2. B cell subsets definitions and phenotypes.

Parental population	Subset definitions and phenotype
**Transitional 1/2**	**Transitional CD21$^{neg/lo}$ (Tr CD21$^{neg/lo}$)**<br>CD19^{pos} CD27^{neg} CD38^{hi} CD24^{hi} CD21$^{neg/lo}$
	**Transitional CD21^{pos} (Tr CD21^{pos})**<br>CD19^{pos} CD27^{neg} CD38^{hi} CD24^{lo} CD21^{pos}
**Naive**	**Resting Naive (resN)**<br>CD19^{pos} CD27^{neg} IgD^{pos} CD38^{pos} CD24^{neg} CD11c^{neg}
	**Activated Naive (actN)**<br>CD19^{pos} CD27^{neg} IgD^{pos} CD38^{neg} CD24^{neg} CD11c^{pos}
**Antibody-Secreting Cells**	**Plasmablasts (ASC)**<br>CD19^{pos} CD27^{hi} CD38^{hi}
**Classic Memory**	**Unswitched Classical Memory (USwM)**<br>CD19^{pos} CD27^{pos} IgD^{pos}
	**Switched Classical Memory (SwM)**<br>CD19^{pos} CD27^{pos} IgD^{neg}
**Non-classic Memory (CD27^{neg})**	**Non-Classic CD27^{neg} IgD^{neg} Memory (M CD27^{neg} IgD^{neg})**<br>CD19^{pos} CD38$^{neg/lo}$ CD24^{pos} CD27^{neg} IgD^{neg}
	**Non-Classic CD27^{neg} IgD^{pos} Memory (M CD27^{neg} IgD^{pos})**<br>CD19^{pos} CD38$^{neg/lo}$ CD24^{pos} CD27^{neg} IgD^{pos}
**Double Negative (CD27^{neg} IgD^{neg})**	**Double Negative 1 (DN1)**<br>CD19^{pos} CD27^{neg} IgD^{neg} CD38$^{neg/pos}$ CD24^{neg} CD21^{pos} CD11c^{neg}
	**Double Negative 2 (DN2)**<br>CD19^{pos} CD27^{neg} IgD^{neg} CD38^{neg} CD24^{neg} CD21^{neg} CD11c^{pos}
	**Double Negative 3 (DN3)**<br>CD19^{pos} CD27^{neg} IgD^{neg} CD38$^{neg/pos}$ CD24^{neg} CD21^{neg} CD11c^{neg}
	**Double Negative 4 (DN4)**<br>CD19^{pos} CD27^{neg} IgD^{neg} CD38^{neg} CD24^{neg} CD21^{pos} CD11c^{pos}

Supplementary Table 3. B cell subsets absolute counts.

B Cell Subset	Control	Mild/Moderate	Severe	Critical	P value (Mild/Moderate vs Severe)	P value (Mild/Moderate vs Critical)
CD19+ B cells count/mL	58,310 (36,971-68,804)	99,810 (54,599-166,600)	73,041 (39,747-108,953)	67,147 (29,062-101,090)	ns	ns
Memory B cells count/mL	20,925 (19,214-25,679)	23,126 (12,956-37,155)	14,258 (6,412-18,904)	17,593 (6,379-24,273)	0.018	0.048
USwM B cells count/mL	2,867 (1,630-3,375)	2,667 (1,821-4,522)	1,030 (510-1,848)	1,316 (606-2,136)	0.001	0.007
SwM B cells count/mL	18,425 (16,125-22,080)	20,328 (10,240-32,953)	11,353 (5,689-16,401)	15,847 (5,655-21,351)	0.02	ns
ASC count/mL	334 (162-437)	1,276 (669-1,725)	1,127 (922-2,781)	909 (296-3,082)	ns	ns
T1/T2 B cells count/mL	697 (544-976)	7,497 (2,065-9,608)	1,674 (602-5,098)	1,433 (473-976)	0.006	0.006
TrCD21neg/lo B cells count/mL	137 (71-297)	2,626 (549-3,503)	810 (304-1,616)	695 (106-3,043)	ns	0.02
TrCD21pos B cells count/mL	571 (461-803)	4,376 (1,312-6,120)	304 (38-1,785)	358 (272-1,981)	0.0002	0.002
Non-Classic Memory count/mL	8,945 (3,013-12,604)	11,578 (9,270-18,929)	12,579 (5,685-16,615)	9,912 (2,678-16,499)	ns	ns
M CD27neg IgDneg count/mL	4,321 (1,956-7,114)	7,691 (6,068-12,274)	7,597 (3,839-1,678)	6,556 (1,496-9,592)	ns	ns
M CD27neg IgDpos count/mL	2,641 (1,054-4,603)	3,838 (2,977-9,169)	3,064 (1,399-5,664)	3,008 (1,338-5,565)	ns	ns
DN B cells	7,706	14,439	10,240	15,919	ns	ns

count/mL	(4,436-10,009)	(7,964-25,352)	(5,605-25,317)	(4,334-19,937)		
DN1 B cells<br>count/mL	3,818<br>(3,235-5,219)	6,414<br>(3,752-11,897)	3,605<br>(1,595-5,127)	3,586<br>(500-6,751)	0.008	0.009
DN2 B cells<br>count/mL	30<br>(14-245)	46<br>(24-98)	82<br>(24-477)	24<br>(0-46)	ns	ns
DN3 B cells<br>count/mL	2,862<br>(1,122-5,580)	5,044<br>(2,992-13,778)	5,962<br>(2,924-20,086)	10,446<br>(2,558-14,506)	ns	ns
DN4 B cells<br>count/mL	4<br>(2-10)	0<br>(0-18)	0<br>(0-14)	0<br>(0-12)	ns	ns
Naive B cells<br>count/mL	12,131<br>(7,814-22,430)	20,113<br>(7,649-56,431)	16,885<br>(7,053-30,327)	13,380<br>(4,928-28,841)	ns	ns
resN B cells<br>count/mL	12,066<br>(7,755-22,361)	17,567<br>(7,649-56,268)	16,761<br>(7,035-30,111)	13,279<br>(4,902-28,748)	ns	ns
actN B cells<br>count/mL	62<br>(32-71)	82<br>(41-333)	128<br>(57-260)	90<br>(9-152)	ns	ns

Data presented are the median (IQR). P values comparing mild/moderate and severe or critical cases were obtained by a Kruskal-Wallis non-parametric ANOVA and Dunn’s uncorrected test. IQR, interquartile range.

Supplementary Table 4. B cell subsets frequency comparisons in age-matched COVID-19 patients vs. healthy subjects.

**B cell subset**	**p-value (Healthy subjects vs COVID-19 patients)**	**Test**
T1/T2 B cells (%)	0.0030**	U
Tr CD21 neg/lo B cells (%)	0.0009***	U
Tr CD21 pos B cells (%)	0.0057**	U
ASC (%)	0.0019**	U
Memory B cells (%)	0.0493*	t
USwM B cells (%)	0.0102*	U
SwM B cells (%)	0.0859^{+}	t
DN1 B cells (%)	0.0617^{+}	U
DN2 B cells (%)	ns	U
DN3 B cells (%)	0.0285*	U
actN B cells (%)	ns	U

*** p < 0.001; ** p < 0.01; * p < 0.05; $^{+}$ p < 0.1

P-values by results of U-Mann-Whitney or t-test for the comparison of the B cell subset frequencies of between healthy controls (n=7) and COVID-19 patients (n=11). All subjects in this analysis are 32-41 years old.

Supplementary Figure 1.

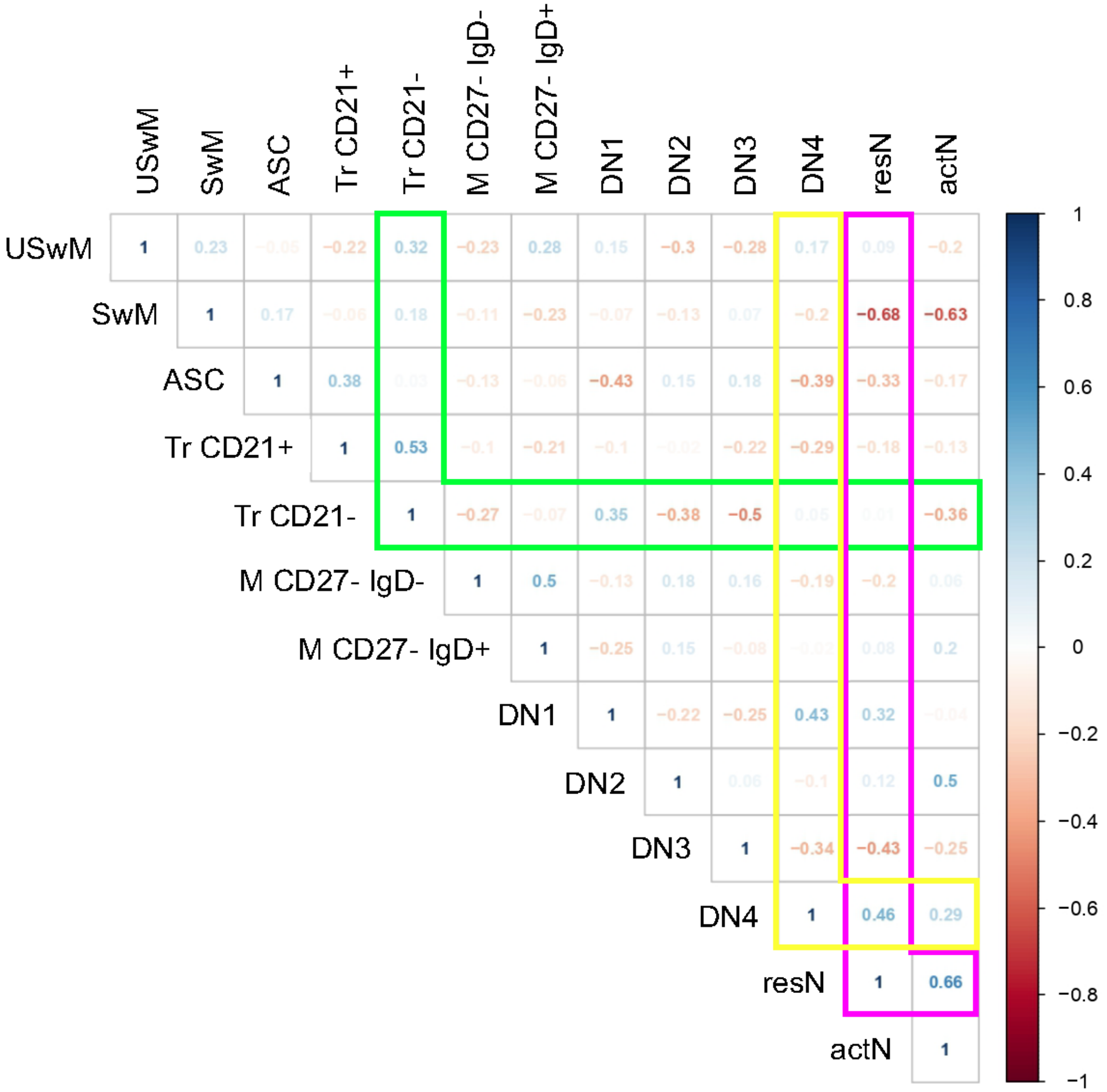

Supplementary Figure 1. Correlation matrix comparing B cell subsets from COVID-19 patients. This matrix comparing all parental-derived B cell subsets, showing the Spearman's correlation coefficient values according to the color scale on the right side; the color intensity of numbers denotes correlation strength. Enclosed values in green, yellow, and magenta indicate a high number of moderately-strong correlations among T CD21^{-}, DN4, and resN, thus making them susceptible to exclusion from hierarchical clustering analysis.
